# Supplementary material for: Bioengineering Human Upper Respiratory Mucosa: A Systematic Review of the State of the Art of Cell Culture Techniques
Source: Bioengineering (Basel). 2024 Aug 13;11(8):826. doi: 10.3390/bioengineering11080826 (PMC11352167; doi:10.3390/bioengineering11080826)
Supplement: Supplementary file 1 [file bioengineering-11-00826-s001.zip › Supplementary_Table_S1_Data_Extraction_Codebook.pdf]

VARIABLE	LABEL	TYPE	DESCRIPTION	CODE	COMMENTS (If missing data report -999)
Study characteristics					
Title	Article title	Alphanumeric	Article title		Report article title
Author	First author	Alphanumeric	Family name of first author		Report only family name of first author (ex: Jack et al)
Year	Year of publication	Numeric	Article year of publication		Report year of publication
Journal	Journal of publication	Alphanumeric	Name of the journal where the article was published		Report journal name
Population characteristics					
Population	Population type	Numeric	Population where the tissue samples were collected from	0 : Pediatric population (age < 18 years old)<br>1 : Adult population (age > 18 years old)<br>2 : Pediatric and adult population<br>3 : Cadaver<br>4 : Not specified	Report code
Sample_size	Sample size	Numeric	Total number of human samples collected for the study		Report sample size
Cell_collection	Method of collection of cells samples	Numeric	Method by which sample was obtained	0 : Bronchial cytology brush<br>1 : Nasal biopsy<br>2 : Nasal brushing<br>3 : Polyp specimens<br>4 : Bronchial biopsy<br>5 : Bronchial cytology brush and nasal biopsy<br>6 : Nasal brushing, polyp specimens and lung explants<br>7: Nasal biopsy, polyp specimens and lung explants<br>8: Lungs explants, nasal brushing	Report code
Cell_line	Anatomical cell lines	Numeric	Cell lines of the samples collected	0 : Bronchial cells<br>1 : Nasal cells<br>2 : Bronchial and nasal cells<br>3: Primary basal epithelial cell	Report code
Cell culture protocol					
Culture_medium	Cell culture medium	Numeric	Medium used for cell culture	0 : Dulbecco's Modified Eagle Medium (DMEM)<br>1 : PneumaCult<br>2 : Bronchial Epithelial Cell Growth Medium (BEGM)<br>3 : DMEM and BEGM<br>4: DMEM, LHC-9<br>5: Rosewell Park Memorial Institute (RPMI) 1640<br>6 : Lonza small airway Epithelial Cell Growth Medium (SAGM)	Report code
ALI_medium	Air-Liquid interface culture medium	Numeric	Medium used for Air-liquid interface cell culture	0 : Dulbecco's Modified Eagle Medium (DMEM)<br>1 : PneumaCult<br>2 : Bronchial Epithelial Cell Growth Medium (BEGM)<br>3: BEGM, AECGM, LHC-8, PneumaCult<br>4 : Bronchial Air-Liquid interface Medium (B-ALI)<br>5 : Bronchial Epithelial Differentiation medium (BEDM)<br>6 : PneumaCult and BEDM	Report code
Antibiotics_medium	Cell culture medium antibiotics	Numeric	Antibiotics supplemented in cell culture medium	0 : Penicillin<br>1 : Streptomycin<br>2 : Gentamicin<br>3 : Vancomycin<br>4 : Tazocilin<br>5 : Colomycin<br>6 : Ciprofloxacin<br>7 : Penicillin and Streptomycin<br>8 : Penicillin, streptomycin and gentamicin<br>9 : Tazocilin, colomycin, ciprofloxacin, penicillin, streptomycin<br>10: Penicillin and gentamicin<br>11: Penicillin, streptomycin, tobramycine, ceftazidim, imipenem –cilastin	Report code
Penicillin_concentration	Penicillin concentration in medium	Numeric	Concentration of penicillin used in cell culture medium		Report value of concentration in U/ml (ex: 0,1)
Streptomycin_concentration	Streptomycin concentration in medium	Numeric	Concentration of streptomycin used in cell culture medium		Report value of concentration in mg/ml (ex: 0,1)
Gentamicin_concentration	Gentamicin concentration in medium	Numeric	Concentration of gentamicin used in cell culture medium		Report value of concentration in mg/ml (ex: 0,1)
Vancomycin_concentration	Vancomycin concentration in medium	Numeric	Concentration of vancomycin used in cell culture medium		Report value of concentration in mg/ml (ex: 0,1)

VARIABLE	LABEL	TYPE	DESCRIPTION	CODE	COMMENTS (If missing data report -999)
Tazocilin_concentration	Tazocilin concentration in medium	Numeric	Concentration of tazocilin used in cell culture medium		Report value of concentration in mg/ml (ex: 0,1)
Colomycin_concentration	Colomycin concentration in medium	Numeric	Concentration of colomycin used in cell culture medium		Report value of concentration in mg/ml (ex: 0,1)
Ciprofloxacin_concentration	Ciprofloxacin concentration in medium	Numeric	Concentration of ciprofloxacin used in cell culture medium		Report value of concentration in mg/ml (ex: 0,1)
Enzyme_digestion	Enzyme used for cell digestion	Numeric	Enzyme used for digesting collected samples	0 : None<br>1 : Collagenase H<br>2 : Pronase<br>3 : DNase<br>4: Protease<br>5: DNase + protéase<br>6: DNA+protéase+pronase	Report code
Time_digestion	Time of cell digestion	Numeric	Total time of cell digestion with the enzyme		Report data in hours (ex: 4,75)
Temperature_digestion	Temperature of cell digestion	Numeric	Temperature at which cells were left for digestionwith enzyme		Report data in degrees celcius (ex: 37,8)
Differentiation_method	Method for cell differenciation	Numeric	Culture method for obtainning differentiated epithelial cells	0 : Air-Liquid Interface culture (ALI)<br>1 : Submerged culture<br>2 : ALI and Submerged culture	Report code
Immunohistochemistry protocol					
Goblet_stain	Goblet cells immunohistochemistry stain	Numeric	Goblet cells identification by immunohistochemistry	0 : None<br>1 : MUC5Ac<br>2 : Periodic acid shiff stain (PAS)	Report code
Ciliated_stain	Ciliated cells immunohistochemistry stain	Numeric	Ciliated cells identification by immunohistochemistry	0 : None<br>1 : B-tubulin<br>2 : Acetylated tubulin<br>3 : Cytokératin-18	Report code
Tight_junction	Tight junction immunohistochemistry stain	Numeric	Tight junction identification by immunohistochemistry	0 : None<br>1 : Zonula-adherence-1 (ZO-1)<br>2 : Fluorescein isothiocyante (FITC)<br>3 : ZO-1 and FITC	Report code
Cell_viability	Cell viability evaluation	Numeric	Evaluation of cell viability	0 : None<br>1 : Lactate Dehydrogenase (LDH)<br>2 : Live-cell propidium iodide (PI)<br>3 : Trypan blueu<br>4 : LDH and PI	Report code
Study findings					
Complete_mucosa	Complete mucosa obtained	Dichotomic	Did the authors obtain a complete manipulable mucosa ?	0 : No<br>1 : Yes	Report code
Mucosa_purpose	Primary use of nasal mucosa by authors	Numeric	Purpose of developping a nasal mucosa	0 : Viral research<br>1 : Pharmacology research<br>2 : Reconstructive Surgical research<br>3 : Cystic fibrosis research<br>4 : Physiology research<br>5 : Protocol description	Report code
TEER_evaluation	ransepithelial transendothelial electrical resistance (TEER) measurement	Dichotomic	Evaluation of tight junction using TEER measurement	0 : Non<br>1 : Yes	Report code
Other					
Supplemental_findings	Other pertinent elements of article	Alphanumeric	Other interesting or pertinent elements of article		Report other interesting findings of article
-777	See supplemental findings				
-888	Not applicable				
-999	Missing data				
