# Supplementary material for: Bioengineering Human Upper Respiratory Mucosa: A Systematic Review of the State of the Art of Cell Culture Techniques
Source: Bioengineering (Basel). 2024 Aug 13;11(8):826. doi: 10.3390/bioengineering11080826 (PMC11352167; doi:10.3390/bioengineering11080826)
Supplement: Supplementary file 1 [file bioengineering-11-00826-s001.zip › Supplementary_Table_S2_Extraction_Data.pdf]

Title	Author	Year	Journal	Population	Sample_size	Cell_collection	Cell_line	Culture_medium	ALI_medium	Antibiotics_medium	Penicillin_concentration	Streptomycin_concentration	Gentamicin_concentration	Vancomycin_concentration	Tazocilin_concentration	Colomycin_concentration	Ciprofloxacin_concentration	Enzyme_digestion	Time_digestion	Temperature_digestion	Differentiation_method	Goblet_stain	Ciliated_stain	Tight_junction	Complete_mucosa	Mucosa_purpose	TEER_evaluation	Cell_viability	Supplemental_findings
Isolation, cultivation, and application of primary respiratory epithelial cells obtained by nasal brushing, polyp samples, or lung explants	Golec et al	2022	STAR Protocols	4	-999	6	2	0	0	9	100	-999	-999	-999	0,01	0,016	0,003	2	12	4	0	-999	-999	-999	0	3	1	0	
A toolbox for studying respiratory viral infections using air-liquid interface cultures of human airway epithelial cells	Michi et al	2021	American Journal of Physiology	3	-777	4	2	1	1	7	100	-777	0,05	-888	-888	-888	-888	2	40	4	0	1	1	2	0	0	0	4	Strep concentration is 100 U/ml<br>Gentamycin was only added in the wash solution<br><br>Sample size not specified : They bought human lungs from a tissue retrieval service<br><br>Cells were immediately frozen after being digested ! Because cells hate serial passing. So you recieve cells, digest them, freeze them, thaw and culture under ALI<br><br>Immunohistochemistry ++++ to assess cell structure and morphology<br><br>Celll viability evaluation<br>- Lactate dehydrogenase : Measures LDH release from necrotic cells<br>- Lice cell propidium iodide : Evaluates necrotic cells
The mystery behind the nostrils – technical clues for successful nasal epithelial cell cultivation	Aydin et al	2021	Annals of Anatomy																										
Isolation, expansion, differentiation, and histological processing of human nasal epithelial cells	Manna et al	2021	STAR Protocols	1	1	2	1	1	1	7	100	0,1	-888	-888	-888	-888	-888	0	-888	-888	0	1	2	0	0	5	0	0	Also evaluated p53 by immunofluoresence
Preliminary Study on the Development of In Vitro Human Respiratory Epithelium Using Collagen Type I Scaffold as a Potential Model for Future Tracheal Tissue Engineering	Lokanathan et al	2021	Applied sciences	4	-999	1	1	-777	-777	-999	-888	-888	-888	-888	-888	-888	-888	1	1	37	0	0	0	0	0	2	0	0	Digestion with collagenase 1<br>Medium: Mixed of DKSFM, F12 and DMEME ratio 2: 1: 1<br><br>Identified in their previous paper nasal cells using : MUC5B, Cytokeratin 14 & 18<br><br>Key notes on developping a function 3D model<br>- A good basal lamina for cell interaction : Collagen in this paper<br>- Mesenchymakl fibroblasts<br>- ALI culture for mucociliary differentiaition<br><br>Many studies support the usage of Retinoic acid as an aid to mucociliary differentiation
Ciliated Epithelial Cell Differentiation at Air-Liquid Interface Using Commercially Available Culture Media	Lee et al	2020	Methods in molecular biology	4	-999	2	1	0	3	7	100	0,1	-888	-888	-888	-888	-888	0	-888	-888	0	1	1	0	0	5	1	0	Co-culture of cells : DMEM + 3T3-J2 (Mouse fibroblasts)<br>Used LHC-9 media (LHC-8 + Retinoic acid + epinephrine)<br><br>Ciliary beat frequency (Hertz) after 28 days in ALI<br><br>Cells survive better in Pneuma-Cult however cillia beating frequency was lower and a higher content in mucus was observed<br><br>No TEER difference was observed
Structural and functional variations in human bronchial epithelial cells cultured in air liquid interface using different growth media	Leung et al	2020	Jounal of Physiology at University of Masscussets	3	6	4	2	2	6	-999	-999	-999	-999	-999	-999	-999	-999	6	16	16	2	1	0	3	0	4	1	0	Objective : Compare histologically nasal cells cultured in ALI Pneuma-cult vs ALI BEDM<br><br>Cell digestion : Protease was first used to digest human airways. Afterwards, Pronase and DNase was used to sepaate epithlium from trachea and bronchi<br>Globlet stain: MUC5AC and MUC2 and MUC5B immunohisto and also expression level via westerne blot<br>Cilia evaluated by ciliary beat and western blot expression level of cilia basal-body protein<br><br>results: Pneumocult -> more ciliated cells and Periodic acid shift (PAS)-positived cells, epithelial thickness taller, increased expression of protein MUC5AC and cilia basal-body protein<br><br>Important that media is supplement with reinoic acid to ensure differentiation of cells<br><br>Poorly differentiated structures are associated with higher TEER... So Pneuma-Cult cultures had lower TEER than BEDM. So well differentiated cells have lower TEER because of the presence of numerous active ion channels<br><br>Conclusion : Pneumacult is better overall
Physical and immunological barrier of human primary nasal epithelial cells from non-allergic and allergic donors	Bergougnan et al	2020	World Allergy Organization	4	-999	5	1	3	0	8	-999	-999	-999	-888	-888	-888	-888	0	-888	-888	0	1	2	1	0	4	1	3	No digestion, sample was just put into trypsin and passaged<br>For ALI, used a mix 1:1 of DMEM and Airway Epithelial Cell Growth medium (Promocell)<br><br>Evaluation of rt-PRC and flow cytometry allergic vs healthy response to stimulation.<br><br>Healthy cells had a rapid growth compared to allergic donors. Also, there was a higher number of viable cells in the healthy population but no difference in the amount od dead cells<br><br>They also used Occludin and E-Cadherin as markers for tight junctions
Different Culture Conditions Affect Drug Transporter Gene Expression, Ultrastructure, and Permeability of Primary Human Nasal Epithelial Cells	Kreft et al	2020	Pharmaceutical Research	4	-999	-777	1	-777	-777	-999	-999	-999	-999	-999	-999	-999	-999	-888	-888	-888	0	0	0	-777	0	2	1	3	Cells were bought from Epithelix Sarl<br>Comparaison of 3 ALI medium and liquid-liquid culture<br>- Epithelix culture medium<br>- PromoCell Airway Epithelial Cell growth medium<br>- Adavanced Minimum Essential Medium (A-MEM)<br><br>Tight junctions were evaluated by : Protein occludin, E-cadherin and Cytokeratin-7<br><br>Cells grown in PromoCell had pleimorphic but numerous microvilli compared to the others<br>Cells culture in Promocell and epithalix resulted in enlarged cells and presence of vacuole -> autosomale/autophagic origine<br>On average ALI -> higher TEER than submerged culture
Utilization of Air–Liquid Interface Cultures as an In Vitro Model to Assess Primary Airway Epithelial Cell Responses to the Type 2 Cytokine Interleukin-13	Everman et al	2018	Methods in molecular biology	4	-999	-999	3	3	1	2	-999	10	-888	-888	-888	-888	-888	-999	-999	-999	0	-999	-999	-999	0	4	0	3	Penicillin concentration 10 000 IU
Air–Liquid Interface Culture of Human and Mouse Airway Epithelial Cells	Jiang et al	2018	Methods in molecular biology	4	-999	4	2	2	2	7	-999	-999	-888	-888	-888	-888	-888	4	12	4	0	-999	-999	-999	0	5	0	0	
Non-woven bilayered biodegradable chitosan-gelatin-polylactide scaffold for bioengineering of tracheal epithelium	Romanova et al	2018	Cell Proliferation-Willey Journal	4	-777	4	2			8	100	1	0,05	-888	-888	-888	-888	4			0					2			Objective : Culture a biodegradable non-woven material in order to obtain a wel-dfferentiated human airway epithelium<br><br>Sample size was not specified
In vitro Methods for the Development and Analysis of Human Primary Airway Epithelia	Gianotti et al	2018	Frontiers in Pharmacology	4	-777	8	1	0	0	7	100	0,1	-888	-888	-888	-888	-888	4	12	4	0	1	1	1	0	3	1	0	Good article for antibiotics for CF epithelia<br>Sample size not specified<br>Good TEER values -> 5 omhs for non CF epithelia<br><br>Transport ions was measured using an usssing chamber<br><br>Cells are cultured in a proliferative medium until fourth passage. (Mix of 1:1 LHC basal medium and RPMI)<br>Whe using a serum free medium they obtained more ciliated and mucus cells<br><br>Rapidly beating cilia meant good cell viability
Characterizing well-differentiated culture of primary human nasal epithelial cells for use in wound healing assays	Schagen et al	2018	Laboratory investigation	1	18	2	1	2	4	-999	-999	-999	-999	-999	-999	-999	-999	-888	-888	-888	0	1	2	1	0	4	1	0	Seed cells at 25 000 cells par insert (Plaque 6 puits)<br><br>Cells were kept in ALI for 7 weeks and TEER was measured at 3, 5 & 7 weeks<br><br>Only 3weeks to observe full differentiation
Characterization of pediatric cystic fibrosis airway epithelial cell cultures at the air-liquid interface obtained by non-invasive nasal cytology brush sampling	Schogler et al	2017	Respiratory Research	0	7	2	1	2	1	-777	-888	-888	-888	-888	-888	-888	-888	-888	-888	-888	0	1	1	1	0	3	1	0	Basal cells was stained using P63<br>Cell layer integrity tested with Fluorescein isothiocyanate-dextran mixed with pneumocult added apicaly and incubated 4h, they evaluated the basal fluorescence after
In Vitro Modeling of RSV Infection and Cytopathogenesis in Well-Differentiated Human Primary Airway Epithelial Cells (WD-PAECs)	Broadbent et al	2016	Methods in molecular biology	0	-999	0	2	0	0	7	100	0,1	-888	-888	-888	-888	-888	-999	-999	-999	2	1	1	1	0	0	1	3	TEER result value should be > 300 ohms / cm2<br><br>If liquid on apical surface of ALI cells = evidence of mucus production. Aspirate to ensure viability of ALI culture
Rapid expansion of human epithelial stem cells suitable for airway tissue engineering	Butler et al	2016	American Journal of Respiratory and critical care Medicine	4	-999	4	1	3	2	8	100	0,1	0,01	-888	-888	-888	-888	-999	-999	-999	0	1	2	0	1	2	1	0	Objective : Design a scalable cell culture system to culture respiratory cells for in vivo grafts<br><br>Use inactivated mouse embryonic fibroblast feeder cells and ROCK inhibition to immortalized epithelial cells and increase proliferation<br><br>Explants cultured in BEGM, co-culture in DMEM, Pen-Stre used GIBCO1570<br><br>Cells grown in combination with 3T3-J2 feeder cells and Y-27632 led to formation of colonies of small epithelial cells whose morphology did not change with passage. Which was not the case with BEGM<br><br>Cells grown in 3T3+Y maintained ciliated differentiation after multiple passage which BEGM did not<br><br>Also, evaluate formation of tracheosphere BEGM vs 3T3-J2 feeder cells and Y-27632<br><br>3 important criteria for and ideal epithelial expansion system<br>- Autologous tissue = no adverse immune response<br>- Rapidly expandable<br>- High in quality in terms of karyotype and phenotype<br><br>They obtained a complete a complete mucosa but it was a Xenograft (Mix of human nasal cells and mouse firboblast) grown afterwards on a decellularized scaffold
A Rotating Bioreactor for Scalable Culture and Differentiation of Respiratory Epithelium	Brickman Raredon et al	2015	Cell Medicine	1	-888	-777	2	6	4	7	-999	-999	-888	-888	-888	-888	-888	-888	-888	-888	0	1	1	1	0	5	0	0	Objective : Culturing cells using a rotating bioreactor model<br><br>Cell line : They bought NHBE cells from Lonza (CC-2540s)<br><br>Utilize anti-pan cytokeratin (sc-8018)<br><br>Advantages of rotating models vs ALI<br>- Medium changes itself continuosly so need to worry about it<br><br>Results: no difference in cell viabilty, but more tight junction, more MUC5AC and ciliated cells in rotating ALI
A Rotating Bioreactor for Scalable Culture and Differentiation of Respiratory Epithelium	Brickman Raredon et al	2015	Cell Medicine	1	-888	-777	2	6	4	7	-999	-999	-888	-888	-888	-888	-888	-888	-888	-888	0	1	1	1	0	5	0	0	Objective : Culturing cells using a rotating bioreactor model<br><br>Cell line : They bought NHBE cells from Lonza (CC-2540s)<br><br>Utilize anti-pan cytokeratin (sc-8018)<br><br>Advantages of rotating models vs ALI<br>- Medium changes itself continuosly so need to worry about it<br><br>Results: no difference in cell viabilty, but more tight junction, more MUC5AC and ciliated cells in rotating ALI
Isolation and culture of primary human nasal epithelial cells from anesthetized nasal epithelia	Hussain et al	2014	Acta oto-laryngologica	1	11	2	1	5	-888	7	-777	-777	-888	-888	-888	-888	-888	0	-888	-888	1	0	0	0	0	1	0	3	Objective : Examine the effect of local anesthesia in patient undergoing nasal brushing to cellect cells<br><br>Utiliseze Cytokeratin-18 (CK-18) to stain for epithelial cells<br><br>Penicillin and streptomycin concentration : 1%
Human Nasal and Tracheo-Bronchial Respiratory Epithelial Cell Culture	Fulcher et al	2013	Methods in molecular biology	4	-999	4	2	2	0	8	100	0,1	0,05	-888	-888	-888	-888	5	24	4	0	-999	-999	-999	0	5	0	0	Penicillin concentration 100 U/ml
Culturing of Human Nasal Epithelial Cells at the Air Liquid Interface	Muller et al	2013	Journal of Visualized Experiments	4	-999	1	1	2	1	-999	-888	-888	-888	-888	-888	-888	-888	3	0,3	-777	0	0	0	0	0	5	0	0	Digestion temperature : Room temperature<br>Taux de succès d'environ 80% pour ce protocol
Comparison of human nasal epithelial cells grown as explant outgrowth cultures or dissociated tissue cultures in vitro	Jiao et al	2013	Higher Education			1	1	0		7	100	0,1	-888	-888	-888	-888	-888	4	12	4			1	1	0	4			Patients included -> chronic rhinosinusite

Title	Author	Year	Journal	Population	Sample_size	Cell_collection	Cell_line	Culture_medium	ALI_medium	Antibiotics_medium	Penicillin_concentration	Streptomycin_concentration	Gentamicin_concentration	Vancomycin_concentration	Tazocilin_concentration	Colomycin_concentration	Ciprofloxacin_concentration	Enzyme_digestion	Time_digestion	Temperature_digestion	Differentiation_method	Goblet_stain	Ciliated_stain	Tight_junction	Complete_mucosa	Mucosa_purpose	TEER_evaluation	Cell_viability	Supplemental_findings
The use of nasal epithelial stem/progenitor cells to produce functioning ciliated cells in vitro	Zhao et al	2012	American Journal of Rhinology and Allergy	1	3	1	1	0	4	7	100	0,025	-888	-888	-888	-888	-888	4	12	4	2	1	1	0	0	4	0	0	Antibiotics: with 100 IU/mL of Antibiotic-Antimycotic solution<br>(Gibco-Invitrogen, Grand Island, NY)<br><br>Digestion : Overnight = 12 hours<br><br>Epithelial cell marker stain : P63 and Ki-67 : Proliferation marker stain
An air-liquid interface culture system for small airway epithelial cells	Damian et al	2011	-888	3	5	4	0	6	4	-999	-999	-999	-999	-999	-999	-999	-999	-999	-999	-999	0	-777	1	1	0	5	1	0	Mucus cells: evaluated mucus production via blue Alcian stain<br>CK-19: specific epithelial marker<br><br>FITC was equally used for cillia
Tissue-Specific Stem Cell Differentiation in an in vitro Airway Modela	Prytherch et al	2011	Macromolecular Biosciences	3	-999	-777	2	2	0	7	10 000	10	-888	-888	-888	-888	-888	-888	-888	-888	0	2	0	1	0	5	1	0	NHBE Cells purchased from Lonza<br>Evaluated also Clara cells via CC10 and basal epithelial cells via p63<br><br>Penicillin concentration : 10 000 U/ml<br><br>Utilize scanning electronic microscopy to visualize cells<br><br>Clara cells are secretory non ciliated cells<br><br>Interesting results ! They were able to identify : Clara cells, non ciliated cells, non mucus secretory epithelial cells
Primary Epithelial Cell Models for Cystic Fibrosis Research	Randell et al	2011	Methods in molecular biology	4	-999	-888	1	2	2	8	100	0,1	0,05	-888	-888	-888	-888	4	24	4	0	0	0	0	0	3	0	0	ALI culture medium : 50:50 mixture of BEGM and DMEM<br><br>Protocols for creation of viral vectors, airway epithelial cells lines, report gene assay in ALI, protein expression or knock down
Air–Liquid Interface Culture of Nasal Epithelial Cells on Denuded Amniotic Membranes	Even-Tzur et al	2010	Cellular and Bioengineering	4	-999	1	1	2	-999	-999	-999	-999	-999	-999	-999	-999	-999	2	-999	-999	0	1	1	0		5	0	0	Objective : To describe a way of culturing nasal epithelial cells on the denuded amniotic membrane as it can serve as an adequate replacement for collagen<br><br>Scaffold = Placenta tissue<br><br>Rationale explaining that is placental membrane contains as much collagen as that of the basal membrane of the nose. Therefore, there is no need to buy a scaffold that has to supplemented with extra collagen nutrients<br><br>AM cultured in MEM supplemented with penicillin 100 U/ml, streptomycin 0,1 mg/ml and gentamycin 0,04 mg/ml.<br><br>Use ELISA for quantification of mucin secretion<br>Results:<br>- Denser cilia and bigger cells on AM scaffold than synthetic<br>- Mucin secretion was constant after 14 days<br>- Actin microfilament of cytoskeleton are implicated in the secretion of mucin<br>- Cells adhesion is better on AM than synthetic. Evaluated via airflow stress mesure by wall shear stresses
Human Nasal Turbinates as a Viable Source of Respiratory Epithelial Cells Using Co-Culture System Versus Dispase Dissociation Technique	Norruddin et al	2007	Laryngoscope	4	6	1	1	0	-888	-999	-888	-888	-888	-888	-888	-888	-888	-777	-777	-777	1	1	3	0	0	2	0	0	Objective : Compare respiratory cells grown using co-culture systems vs dispase dissociation techniques... Collagenase vs Dispase<br><br>Comparaison of extraction with collagenase 1 (12h at 4) vs dispase (20-30h at 4)<br>Use a combination of DMEM and DKSFM<br>Cell types evaluated by PCR<br><br>MUC5B gene was also investigated<br><br>Dispase 2 = Protéase !<br><br>Results :<br>- MUC5B showed poor to zero expression in both cultures<br>- More basal cells in the collagenase culture<br><br>- CK-18 a marker for ciliated cell stain was absent and it makes sense because cells were not grown in ALI... So there was no environmental pressure to produce cilia to clear mucosal apical surface
Physiological 3D tissue model of the airway wall and mucosa	Choe et al	2006	Nature protocols	4	-888	-777	0	2	2	7	10 000	10	0,05	-888	-888	-888	-888	0	-888	-888	0	-999	-999	-999	0	5	0	0	Cilia were observed after 3 weeks in ALI<br><br>Model is not designed for in vivo grafts or TEER evaluation<br><br>Use BEBM medium for expansion<br><br>It takes 7 days for ALI cells to differentiate and phenotype was maintained till up to 4 weeks !
Expansion of cultures of human tracheal epithelium with maintenance of differentiated structure and function	Widdicombe et al	2005	BioTechniques	3	3	4	2	0	-888	8	100	0,1	0,05	-888	-888	-888	-888	4	12	4	1	-999	-999	-999	0	5	1	0	Objective : Develop a simple passagin technique<br>20 000 cellules par T25 & 500 000 cellules par T75<br><br>Loss of differentiation after 3 passages. So cells at P3, P4 are well differentiated !<br>However, cilia developps faster when cells are at P0 and P1<br><br>Media supplemented with LHC-9 : Lechne's laboratory of human carcinogenesis basal medium 9<br><br>Digestion time : Overnight = 12 hours
Isolation and air–liquid interface culture of human large airway and bronchiolar epithelial cells	Bals et al	2004	Journal of Cystic Fibrosis	4	-777	4	2	0	0	11	50	0,05	-888	-888	-888	-888	-888	4	24	4	0	-999	-999	-999	0	3	1	0	Antibiotics concentration<br>- Imipenem-cilastin : 0,05 mg/ml<br>- Ceftazidine : 0,05 mg/ml<br><br>Sample size was not reported<br><br>TEER measurement should be followed using an ohmmeter
Production Of Tissue-Engineered Three-Dimensional Human Bronchial Models	Paquette et al	2003	In Vitro Cellular and Developmental Biology	4	-999	0	0	0	0	10	100	-888	0,025	-888	-888	-888	-888	1	-999	-999	0	2	0	0	0	5	0	0	Utilizie cytokeratin-14 (CK-14) and epithelial keratin A-E3<br><br>Identification of mucoglycoproteins using periodic acid shiff stain
Engineering a Composite Neotrachea With Surgical Adhesives	Doolin et al	2002	Journal of Pediatric Surgery			-777		2																		2			
Living epithelial-mesenchymal compounds formed in vitro suitable for autografting	Ross et al	1997	European Archives of Oto-Rhino-Laryngology	4	8	2	1	0	-888	7	100	0,1	-888	-888	-888	-888	-888	2	16	4	1	0	0	0	0	2	0	0	Results: 87% success rate for culture<br>respiratory epithelial cultures cilia<br>disappeared within 2-3 weeks and initial beat frequency<br>was about one-third of normal.
Development of a Human Nasal Epithelial Cell Culture Model and its suitability for transport and metabolism studies under in vitro conditions	Werner et al	1995	Pharmaceutical Research	4	-777	1	1	0	-888	7	100	0,1	-888	-888	-888	-888	-888	4	20	4	1	0	0	2	0	1	0	0	Sample size not specified<br>Digestion time : 16 to 20 hours<br><br>Turbinates were prefered compared to tonsils because tonsils always had way more infections<br><br>Dome formation caused by secretion of solutes and water through basolateral membrane werre an indication of tight junction formation<br><br>Tight junction were also evaluated using FITC-labeled phalloidin<br><br>Only 5% of their monolayer had ciliated cells
Human Respiratory Mucosa in a Non-adshesive Stationary Organ culture System	Steinsvag et al	1991	Laryngoscope	0	45	1	1	2	-888	7	100	0,1	-888	-888	-888	-888	-888	-999	-999	-999	1	2	0	0	0	5	0	-777	Cells were purchased from clonetics corporation<br>Viability: cells considered viable when motile cilia were observed<br><br>They relied on microscopy for cell characteristics identification
A Serum-Free Method For Culturing Normal Human Bronchial Epithelial Cells At Clonal Density	Lechner et al	1985	Journal of Tissue Culture Methods	3	-999	4	0	4	-888	2	-888	-888	0,05	-888	-888	-888	-888	-999	-999	-999	1	0	0	0	0	5	0	0	Medium culture: Three different nutrient media are used, L15,<br>CMRL 1066, and LHC basal. NO DMEM<br>No extraction, just put the explants in the petri
Polarized Cultures Of Human Airway Epithelium From Nasal Scrapings And Bronchial Brushings																													
Regenerative Potential of Human Airway Stem Cells in Lung Epithelial Engineering																													
